# The impact of using community home-based elderly care services on older adults’ self-reported health: fresh evidence from China

**DOI:** 10.3389/fpubh.2023.1257463

**Published:** 2023-09-20

**Authors:** Yang He, Baojian Wei, Yushang Li

**Affiliations:** ^1^School of Business, Xiangtan University, Xiangtan, Hunan, China; ^2^School of Nursing, Shandong First Medical University & Shandong Academy of Medical Sciences, Taian, Shandong, China

**Keywords:** community home-based elderly care services, older adult’s health, public health, influential mechanisms analysis, 2SLS

## Abstract

**Background:**

The rapid population aging in China, characterized by a higher prevalence of illnesses, earlier onset of diseases, and longer durations of living with ailments, substantially engenders challenges within the domain of older adults’ healthcare. Community home-based elderly care services (CHECS) are a feasible solution to solve the problem of older adults’ care and protect older adults’ health. The aim of this study is to investigate the relationship, heterogeneity effects and influential mechanisms between older adults’ use of CHECS and their self- reported health.

**Methods:**

The study employs the Instrumental Variable technique and empirically investigates the relationship, heterogeneity effects and influential mechanisms between older adults using CHECS and their self-reported health using data from the China Longitudinal Aging Social Survey from 2018.

**Results:**

The findings indicate, firstly, that using CHECS considerably improves older adults’ self-reported health. Secondly, the heterogeneity test reveals that the effect is more pronounced for older adults who are under the age of 80, have functional disabilities, are free of chronic diseases, have never attended school, reside in lower-income households, are single, rarely interact with their children, and live in central urban or city/county regions. Thirdly, the mechanism test reveals that the “social network effect” and “family care effect” are the key influence channels of using CHECS.

**Conclusion:**

An empirical foundation for the policy reform of community home-based care for seniors is provided by this study with the limitations to discuss the other socioeconomic aspects such as government health expenditure and discuss the specific services aspects such as health care. The findings carry substantial implications for improving the health of older individuals and provide suggestions for establishing a socialized aged care system in China.

## 1. Introduction

Ageing has become an unavoidable trend in the world due to rising life expectancy and a declining birth rate (1), hence older adults’ care and older adults’ health are growing global problems. The world’s greatest old population and fastest aging population is in China. China had 20.56 million elderly people aged 65 and older by the end of 2021, making up 14.2% of the nation’s overall population (2) and 26.84% of the world’s elderly population (3).

But China’s system for long-term care is in serious trouble. On the one hand, the amount of family care for older adults is declining due to the rapid changes in population structure and the social economy, which indicates that the traditional model of eldercare has not been able to adequately satisfy the expanding demands of older adults (4, 5). On the other hand, there is still a sizable disparity between the supply and demand of elderly care services in the official elderly care market (6). As a result, the Chinese government has been looking at ways to create a system of social old care services that is effective and sustainable in recent years.

Community is the basic unit of social governance (7). The “aging-in-place” preference of older individuals can be satisfied using a community home-based elderly care model, which also successfully relieves the stress of family elder care, lowers the cost of long-term care, and lessens the financial load on the government (8). Community home-based elderly care services (CHECS) have become more significant in the social aged care service system in recent years. On the document, “Outline of the 14th Five-Year Plan (2021–2025) for National Economic and Social Development and Vision 2035 of the People’s Republic of China” proposed to “improve the network of community-based, at-home elderly care services,” and “support idle resources for community-embedded elderly care” (9). In practice, from 2016 to 2020, the central lottery public welfare fund invested 5 billion yuan to carry out pilot reform of community home-based elderly care services in batches (10).

Uncertainty persists on whether using CHECS improves health outcomes for the elderly population. On the one hand, a substantial body of evidence demonstrates that CHECS can enhance older health by fostering a sense of community and maintaining social networks through aging-in-place (11–13). On the other hand, compared to nursing home care, there is often less control of the quality and quantity of home-based care (14, 15). Additionally, low-quality CHECS may increase elderly patients’ medical problems and depression (16). Therefore, it is necessary to accurately estimate the impact of using CHECS on the health of older adults, identify its heterogeneous effect in different populations, and analyze the influence mechanism. This is of great significance to evaluate the effect of the current CHECS, promote the realization of active aging, and improve the “elderly care service system based on the home, supported by the community, supplemented by institutions, and combining medical care with elderly care”.

Previous research examined at CHECS’s impact on older adults’ health from a variety of angles. Three or more things need to be improved (17). In terms of research techniques, the first is. To address the endogeneity issues brought on by sample self-selection, the propensity score matching method was adopted in the majority of earlier investigations (17). However, it is challenging to apply this approach to address the endogeneity issues brought on by missing variables and reverse causality. The second consideration is from the perspective of the research. The majority of studies solely looked at how the perceived availability of CHECS affected older adults’ health. Therefore, it is essentially unknown how using these services may affect older adults’ health. The third aspect is in terms of research content. We examined the possible heterogeneous effects of using CHECS from multiple perspectives. In addition, we also empirically examine the possible influencing mechanism. Existing literature points out that the development of CHECS can not only provide older adults with more ways to meet the basic needs of daily life and better care services, but also significantly reduce the pressure of care within the family (18). So, using CHECS not only helps to prevent older adults from falling into social isolation, and improve their sense of self-efficacy (19, 20), but also helps to improve the physical and mental health of caregivers, reduce the frequency of negative manifestations such as impatience and elder abuse, and improve the quality of caregivers’ care for older adults (21, 22).

This work develops a 2SLS model and uses “the perceived rate of CHECS at community level” and “except for individuals, the utilization rate of CHECS at community level” as instrumental variables based on data from the China Longitudinal Aging Social Survey (CLASS) in 2018. The goal of this work is to investigate the impact of using CHECS on the self-reported health of Chinese older adults. Additionally, we conducted an empirical analysis of the impact of using CHECS on the respondents’ self-reported health in various groups, taking into account the respondents’ age, physical limitations, chronic illnesses, education, *per capita* household income, living arrangements, emotional support from their families, and living region (community location). The results are helpful for expanding CHECS’s area of study on the health of Chinese older people. We seek to more fully grasp the impact of the current CHECS and offer a crucial practical guide to further optimize CHECS supply, illuminating the execution of future policies on the growth of CHECS in China.

## 2. Materials and methods

### 2.1. Data

The cross-sectional data from the China Longitudinal Aging Social Survey (CLASS), conducted in 2018 by the National Survey Research Center at Renmin University of China (NSRC), served as the basis for this study’s analysis. A nationally representative sample of adults over 60 was chosen for the survey using a stratified, multi-stage, probabilistic sampling technique. Within this methodology, a nationally representative sample of 11,419 participants was selected from 28 provinces, excluding Hong Kong, Taiwan, Macau, Hainan, Xinjiang, and Tibet. Highly trained interviewers conducted face-to-face interviews to systematically and comprehensively collect information pertaining to the essential attributes, physical and mental well-being, lifestyle choices, intergenerational relationships, long-term care service necessities, and social support resources of older participants. The CLASS dataset for 2018 consisted of a total of 809 variables. The survey’s primary objectives were to assess the basic conditions, current situation, personality and emotional traits, lifestyle, activities of daily living (ADL), instrumental activities of daily living (IADL), personal background, family structure, and level of physical health of older adults. For this study’s data, a survey conducted in 2018 questioned about the availability of community home-based elderly care services (CHECS) and how often older adults used them. The total number of valid samples was 9,654 after accounting for unreachable and missing values for important variables. Throughout the entire interviewing process, participants remained anonymous and voluntary.

### 2.2. Variable

#### 2.2.1. Dependent variable

The question, “In overall, how do you rate your health?” is posed to CLASS respondents. The following categories of responses are possible: very good, good, fair, poor, and very poor. To create indicator variables for very good or good health, we recode this variable.

#### 2.2.2. Independent variables

CLASS respondents are asked, “Whether you have used the social services provided by the community.” CHECS includes door-to-door visit, senior citizen service hotline, visiting the doctor, daily shopping, providing legal aid, daily care, deliver meals to older adults, day activity center and spiritual comfort. The possible response categories are “yes” and “no.” If all the services were not utilized, independent variables “UCHECS” were assigned a value of 0.

#### 2.2.3. Instrumental variable

The self-reported health of older adults may be affected by unobserved factors, such as the price and quality of CHECS (23), which may relate to the use of CHECS by older adults (24). In addition, a possible reverse causation between CHECS utilization and self-reported health: older people with poorer health are more likely to use CHECS (25). The OLS model exhibits significant endogeneity as a result of these issues. Therefore, to find the true causal effects, we employed IV estimation. We used “the perceived rate of CHECS at community level” (CP_CHECS) and “except for individuals, the utilization rate of CHECS at community level (CU_CHECS)” as instrumental variables. CP_CHECS was measured by the percentage of older people in the community who knew that the community provides CHECS. CU_CHECS was measured by excepting for individuals, the percentage of older people in the community who used CHECS. These two variables meet two fundamental criteria for being used as instrumental variables. First, as mentioned in Bakeera et al. (26) and peer effects, CP_CHECS and CU_CHECS have an important influence on the usage of CHECS by older adults. Second, the characteristics of CHECS at the community level do not correlate with the hidden variables influencing older individuals’ self-reported health. Only its impact on the usage of CHECS can have an impact on the self-reported health of older adults.

#### 2.2.4. Control variables

In order to apply controls on the impact of other factors on self-reported health of older people, we introduced 17 variables at the individual, family, and community levels.

Four different types of control variables were established at an individual level, including physical health levels, socioeconomic status, natural features, and health behaviors. Age, the logarithm of each person’s annual wage, and the number of cohabitants are all examples of continuous variables. All of the following dichotomous variables have values of 1 for females and 0 for males, 1 for non-agricultural and 0 for agricultural, 1 for being married and 0 for everyone else, 1 for having commercial insurance or social security and 0 for not, and 1 for having chronic illness and 0 for not. Additionally, gender, household registration, marital status, whether or not one smokes, whether or not one has chronic illness, and whether or not one is married are all dichotomous variables. Education is coded as dummy variables out of four categories: never received education (omitted group), primary school, junior high school, senior high school or above. Daily activities include 2 different activity types – ADL and IADL, which is coded as dummy variables out of four categories: limitations in neither ADL nor IADL (omitted group), limitations in ADL, limitations in IADL, limitations in both ADL and IADL. ADL including “dressing, bathing, eating, walking indoors, going to the toilet, and bowel control,” IADL including “going up and down stairs, walking outdoors, using transportation, shopping, managing finance, carrying goods, preparing meals, and housecleaning.” In the CLASS questionnaire, interviewees were asked whether they could independently complete 6 ADL and 8 IADL, if any one of the activities cannot be completed independently, we identified the respondents as ADL/IADL limited.

In terms of family level, we introduced four characteristics: *per capita* household income, number of surviving children, family financial support, and family emotional support. The logarithm of *per capita* household income and number of surviving children are continuous variables. In the CLASS questionnaire, for every surviving child, the interviewee was asked “How much cash, food, or gifts did this child give you in the past year?” and “How often did you meet up with this child in the past year.” Based on the first question, we constructed the variable “family financial support.” If any one of the children gave cash, food, or gifts to the respondent, we assigned the value of 1, otherwise, the variable “family financial support” was assigned a value of 0. Based on the second question, we constructed the variable “family emotional support.” If the respondents saw any of their children at least once a month, then the family was considered to provide emotional support and was assigned a value of 1. Otherwise, it was assigned a value of 0.

Finally, we also introduced two community characteristics: whether having senior centers and other facilities in community, and community location. In the CLASS questionnaire, respondents were asked “Does your community have any of the following activities or facilities.” The possible response categories are: senior citizens activity center, fitness centers/facilities, chess room, reading room, outdoor playgrounds, other else, and none of the above. If the respondent answered “none of the above,” then the community was considered not to provide activity place or facility for older adults and was assigned a value of 0. Otherwise, it was assigned a value of 1. The variable “community location” was constructed based on the question “Which type of area respondents live in?.” If the community was in the central urban region of city/county, the variable “community location” was assigned a value of 1. Otherwise, it was assigned a value of 0.

#### 2.2.5. Mechanism variables

We examined the mechanisms from two perspectives: family care and social networks. Social isolation and self-efficacy are the two factors used in this study to measure the social network. In order to quantify social isolation on a multidimensional level, this work chooses three indicators: emotional support, social adaptability, and perception of the social environment. Based on the inquiry, “Have you felt unaccompanied for the past week?” we created the variable “emotional support.” Older adults were regarded to be receiving emotional support if the respondents said “no,” and a value of 1 was assigned. If the respondent answered “sometimes” or “often,” it was assigned a value of 0. We constructed the variable “social adaptation” based on the question “Society is changing so fast that it is difficult for me to adapt to the change.” If the respondents answered “fully disagree” or “relatively disagree” or “between relatively disagree and relatively agree,” then the older adult was considered more likely to adapt to the current social changes and was assigned a value of 1. If the respondents answered “relatively agree” or “fully agree,” it was assigned a value of 0. We constructed the variable “social environment perception” based on the question “Current social changes are more and more detrimental to older people.” If the respondents answered “fully disagree” or “relatively disagree” or “between relatively disagree and relatively agree,” then the older adult was considered more likely to believe that changes in the current social environment are not disadvantageous to older adults and was assigned a value of 1. If the respondents answered “relatively agree” or “fully agree,” it was assigned a value of 0. For self-efficacy, based on the question “Have you felt useless in the past week?.” It was assigned a value of 1 for the respondents who answered “no,” and 0 for the respondents who answered “sometimes” or “often.” This work measures family care according to the question “Whether you feel this child does not care enough for you?,” CLASS asked this question to respondents about each surviving child. If one of the children answered “occasionally,” “sometimes” or “often,” the family was considered to have provided enough care for older adults. Otherwise, it was assigned a value of 0.

#### 2.2.6. Descriptive statistics

Table 1 shows the descriptive statistics of the main variables. The mean value of self-reported health among the survey older adults was 0.432. This indicates that 43.2% of older people think they are in good physical health. In addition, we also found that for older adults, there was a significant gap between the use of CHECS and the perception of CHECS: 21.61% of older adults knew that the community provided elderly care services, but only 10.05% of older adults used them. Therefore, the actual coverage rate and effective utilization rate of CHECS are at a low level. 86.97% of Chinese older adults received financial support from their children, this demonstrates that family elderly care is still an important way of aging for older adults in China.

**Table 1:** Descriptive statistics.

Variables’ categorization	Variable	Mean	SD	Min	Max
Dependent variables	Self-reported health	0.432	0.495	0	1
Independent variables	UCHECS	0.100	0.301	0	1
Instrumental variable	CP_CHECS	0.216	0.280	0	1
	CU_CHECS	0.101	0.173	0	1
Mechanism variables	Emotional support	0.491	0.500	0	1
	Social adaptation	0.699	0.459	0	1
	Social environment perception	0.730	0.444	0	1
	Self-efficacy	0.443	0.497	0	1
	Family care	0.809	0.393	0	1
Individual level variables	Gender	0.496	0.500	0	1
	Age	71.813	7.290	62	108
	Household registration	0.466	0.499	0	1
	Marital status	0.685	0.465	0	1
	Never received education	0.273	0.446	0	1
	Primary school	0.410	0.492	0	1
	Junior high school	0.230	0.421	0	1
	High school or above	0.087	0.282	0	1
	Number of cohabitants	1.614	1.230	0	7
	Ln (individual annual income)	9193.771	12531.690	100	60000
	Whether having commercial insurance or social security	0.748	0.434	0	1
	Whether smoking	0.285	0.452	0	1
	Daily activities: limitations in neither ADL nor IADL	0.721	0.448	0	1
	Daily activities: limitations in ADL	0.191	0.393	0	1
	Daily activities: limitations in IADL	0.014	0.116	0	1
	Daily activities: limitations in both ADL and IADL	0.074	0.263	0	1
	Whether having chronic illness	0.723	0.448	0	1
Family level variables	Ln (per capita household income)	11677.990	11161.200	333.3333	55000
	Number of surviving children	2.644	1.369	1	10
	Family financial support	0.870	0.337	0	1
	Family emotional support	0.850	0.357	0	1
Community level variables	Whether having senior centers and other facilities in community	0.666	0.472	0	1
	Community location	0.366	0.482	0	1

Authors’ own calculations.

### 2.3. Empirical models

In order to test the impact of using CHECS on older adults health, the following benchmark regression model is constructed:


(1) $$SRH_{ic}=\alpha_{0}+\alpha_{1}{\mathrm{UCHECS}}_{ic}+\alpha_{2}X_{ic}+\mu_{ic}$$

where the subscript i represents the individual code, subscript c represents the community code. SRH represents the dependent variable indicating the self-reported health of respondent i who lives in community c. UCHECS indicates whether individual i living in community c used CHECS. ***X_ic_*** indicates other demographic control variables and social environmental variables that may affect individual health.

In the OLS model, the key coefficient α_1_ may be biased, because the error term *μ_ic_* may contain unobserved factors related to CHECS utilization and resident self-reported health, and a possible reverse causation between CHECS utilization and self-reported health (27).

To address this endogeneity problem, we referred to Lin et al. (28), which used aggregation data at higher levels as instrumental variables of independent variables at lower levels. and constructed a two-stage least-squares (2SLS) model with “the perceived rate of CHECS at the community level” and “the utilization rate of CHECS at the community level (except for individuals)” as instrumental variables. Equations 2, 3 represent first-stage and second-stage regressions, respectively.


(2) $$\begin{array}{l} {\mathrm{UCHECS}}_{ic}=\beta_{0}+\beta_{1}CP:{\mathrm{CHECS}}_{c}+\beta_{2}CU:{\mathrm{CHECS}}_{c}\\ \;+\beta_{3}X_{ic}+w_{ic} \end{array}$$

(3) $$SRH_{ic}=\gamma_{0}+\gamma_{1}\hat{{\mathrm{UCHECS}}_{ic}}+\gamma_{2}X_{ic}+\varepsilon_{ic}$$

where CP_CHECS indexes the percentage of older people in community c who knew that the community provides CHECS. CU_CHECS indexes except for individuals, the percentage of older people in community c who using CHECS. UCHÊCS*_ic_* is the fitted value of in the first-stage regression. *w_ic_* and *ε_ic_* are the error terms. ***X_ic_*** is defined in the same way as in Equation 1. The IV (2SLS) estimators of coefficients *β*_1_ and *β*_2_ capture the causal effects of UCHECS*_ic_* on SRH*_ic_*, and are the central interest of our research.

## 3. Results

### 3.1. Baseline model regression

The baseline model regression results are shown in Table 2. The results in Columns (1) and (2) show that there is a strong association between CHECS and resident self-reported health in our sample. Further, the columns (3) and (4) results show that 2SLS estimations are identical to this, except the coefficient grows and the significance level declines. In our preferred specification in Column (4), older adults using CHECS increased the probability of assessing their health as good or very good by 16.1 percentage points. The coefficients of “The perceived rate of CHECS” and “Except for individuals, the utilization rate of CHECS” are significant at least at the 10% level in first-stage regression, and the Kleibergen-Paap rk Wald F-statistic is much larger than its critical value, which can exclude the possibility of weak IVs; the Hansen J-test *p*-value is greater than 0.1, which indicates that the null hypotheses cannot be rejected. That provides suggestive evidence that the variables are valid instruments (29). Comparatively, we discovered that the OLS estimates are in fact biased and that putative endogeneity tends to underestimate the beneficial effects of CHECS use on self-reported health. This finding suggests that the OLS model leaves out some unobserved characteristics, like family members’ health, which is inversely associated to the usage of CHECS and positively related to self-reported health.

**Table 2:** Baseline model regression results.

	OLS	OLS	2SLS	2SLS
	(1)	(2)	(3)	(4)
Panel A:2SLS results				
UCHECS	0.036** (0.017)	0.081*** (0.030)	0.156** (0.067)	0.161** (0.074)
Gender		−0.034** (0.014)		−0.033** (0.014)
Age		−0.003*** (0.001)		−0.003*** (0.001)
Household registration		−0.063*** (0.022)		−0.064*** (0.022)
Marital status		0.006 (0.014)		0.005 (0.014)
Education: primary school		−0.036** (0.018)		−0.034* (0.018)
Education: junior high school		0.005 (0.022)		0.007 (0.022)
Education: high school or above		0.083*** (0.028)		0.084*** (0.028)
Number of cohabitants		−0.004 (0.008)		−0.003 (0.008)
Ln (individual annual income)		0.030*** (0.009)		0.031*** (0.009)
Whether having commercial insurance or social security		0.036 (0.022)		0.035 (0.022)
Whether smoking		−0.021 (0.018)		−0.020 (0.018)
Daily activities: limitations in ADL		−0.161*** (0.018)		−0.167*** (0.018)
Daily activities: limitations in IADL		−0.142*** (0.051)		−0.147*** (0.051)
Daily activities: limitations in both ADL and IADL		−0.293*** (0.025)		−0.305*** (0.025)
Whether having chronic illness		−0.202*** (0.019)		−0.203*** (0.019)
Ln (per capita household annually income)		0.005 (0.010)		0.004 (0.010)
Number of surviving children		−0.008 (0.006)		−0.007 (0.006)
Family financial support		−0.030 (0.024)		−0.029 (0.024)
Family emotional support		0.043* (0.022)		0.041* (0.022)
Whether having senior centers and other facilities in community		0.055** (0.022)		0.050** (0.022)
Community location		−0.055** (0.025)		−0.056** (0.025)
Panel B: First-stage results				
CP_CHECS			0.083*** (0.010)	0.039** (0.015)
CU_CHECS			0.801*** (0.021)	0.859*** (0.032)
1st Kleibergen-Paap rk Wald F statistic			1932.601	566.867
Hansen J-test *p*-value			0.896	0.428
Observations	9,654	6,786	9,652	6,785

***Significant at the 1% level; **Significant at the 5% level; *Significant at the 10% level. Robust Standard errors are reported in parentheses in columns (1) and (3); Standard errors clustered at the community level are reported in parentheses in columns (2) and (4).

### 3.2. Robustness test

The above analysis confirms that CHECS had a significant positive impact on the self-reported health of Chinese older population. To verify the reliability of this conclusion, we conducted a series of robustness tests. The results are shown in Table 3.

**Table 3:** Robustness tests.

	Excluding communities with sample sizes of Less than 5	Excluding communities with sample sizes of less than 10	Using "compared with last year, whether the health has improved" as dependent variable
	(1)	(2)	(3)
UCHECS	0.160** (0.074)	0.160** (0.074)	0.045* (0.025)
Control variables	Yes	Yes	Yes
1st Kleibergen-Paap rk Wald F statistic	565.821	558.956	577.167
Hansen J-test *p*-value	0.435	0.420	0.389
Observations	6,777	6,762	6,771

**Significant at the 5% level; *Significant at the 10% level. Standard errors clustered at the community level are reported in parentheses. We included all control variables in column 4 of Table 2.

Firstly, excluding possible outliers. The instrumental variables used in this paper are constructed at the community level, so too small sample size in the community may produce biased estimators. In order to solve this problem, we will exclude communities with sample sizes of less than 5 and 10. The result is quite close to that of our baseline regression.

Secondly, change the dependent variable. Another potential concern is the omitted variable bias. In order to solve this problem, we will use “compared to the last year, whether the health has improved” as the new dependent variable. The direction of the estimated coefficient is the same as that of the baseline regression.

### 3.3. Heterogeneity analysis

Due to the various individual features and care requirements, the demand for CHECS varies greatly for older adults at the period of life decline. Furthermore, the development of CHECS in China differs significantly between urban and rural locations. Daily life support, medical care, and health services are more readily available in urban areas where the CHECS system is more complete (30). In order to determine whether the projected impact varied with the respondents’ age, physical limitations, chronic illnesses, education, *per capita* household income, housing arrangements, family emotional support, and living region (community location), we conducted eight sets of stratified analyses. Table 4 provides a summary of the findings.

**Table 4:** Heterogeneity analysis.

	(1)	(2)
	People aged under 80	People aged 80 or above
Panel A: regression of subsamples by age		
UCHECS	0.121 (0.075)	0.354*** (0.122)
1st Kleibergen-Paap rk Wald F statistic	441.076	54.131
Hansen J-test *p*-value	0.226	0.098
Observations	5,707	1,078

	People without physical limitations	People with physical limitations
Panel B: regression of subsamples by physical limitation		
UCHECS	0.078 (0.079)	0.280*** (0.081)
1st Kleibergen-Paap rk Wald F statistic	258.608	235.396
Hansen J-test *p*-value	0.316	0.669
Observations	5,151	1,634

	People without chronic illness	People with chronic illness
Panel C: regression of subsamples by chronic illness		
UCHECS	0.346** (0.155)	0.120 (0.074)
1st Kleibergen-Paap rk Wald F statistic	48.623	560.674
Hansen J-test *p*-value	0.605	0.463
Observations	1,689	5,096

	Never received education	Primary school and above
Panel D: Regression of subsamples by education		
UCHECS	0.398*** (0.086)	0.077 (0.075)
1st Kleibergen-Paap rk Wald F statistic	128.886	336.124
Hansen J-test *p*-value	0.118	0.181
Observations	1,641	5,144

	Per capita household income above average	Per capita household income less than or equal to average
Panel E: regression of subsamples by per capita household income		
UCHECS	0.018 (0.093)	0.246*** (0.083)
1st Kleibergen-Paap rk Wald F statistic	183.096	321.893
Hansen J-test *p*-value	0.294	0.654
Observations	2,829	3,956

	Living with other peoples	Living alone
Panel F: regression of subsamples by living arrangements		
UCHECS	0.143* (0.074)	0.270** (0.120)
1st Kleibergen-Paap rk Wald F statistic	476.816	61.675
Hansen J-test *p*-value	0.361	0.811
Observations	6,002	783

	Meeting with children every month	Meeting with children not every month
Panel G: regression of subsamples by family emotional support		
UCHECS	0.141* (0.072)	0.277** (0.130)
1st Kleibergen-Paap rk Wald F statistic	440.683	48.574
Hansen J-test *p*-value	0.196	0.122
Observations	5,903	882

	The central urban region of city/county	Other regions
Panel H: regression of subsamples by community location		
UCHECS	0.192** (0.086)	0.126 (0.101)
1st Kleibergen-Paap rk Wald F statistic	338.273	274.302
Hansen J-test *p*-value	0.459	0.492
Observations	2,643	4,142

***Significant at the 1% level; **Significant at the 5% level; *Significant at the 10% level. Standard errors clustered at the community level are reported in parentheses. We included all control variables in column 4 of Table 2.

The results in Panel A and Panel B show that the impact was more evident for people aged 80 or above and people with ADL or IADL difficulties. This may be due to the fact that people who are older and have limited physical abilities usually have poorer physical function, and the informal care has not been able to address significantly the needs of them (31). Therefore, they have a greater need for CHECS to meet their basic needs (32).

We can see from Panel C that using CHECS had a significantly stronger impact on the self-reported health of people without chronic illness. Older adults with chronic illness are more likely to require medical care services and have higher requirements for medical skills and improvisational abilities for service personnel. However, in China, CHECS suffer from lack of qualified LTC professionals, limited service/low service quality, and unrealized integrated care (33).

We can see in Panel D and Panel E that using CHECS had a more positive impact on the self-reported health of people who have never received education and from low-income families. This may be because these older people are more likely to be in a disadvantaged position and have lower requirements for their life quality and elderly care services (34).

We can see according to Panel F and Panel G that for Chinese older adults living alone and having less family emotional support, the greater positive effect of using CHECS on their self-reported health. This may be due to the fact that people who live alone or do not have frequent contact with their children need more formal care from the society to meet daily life needs, and need more external companionship to fulfill emotional needs (35).

Panel H shows the estimated coefficient is higher in the central urban region of city/county regions than other regions, because the economic strength of the region and the government’s financial resources caused by the urban and rural two-dimensional structure lead to a lower level of infrastructure of social support and services for older adults in rural regions (36).

### 3.4. Influential mechanisms

The aforementioned findings show that using CHECS had a considerable impact on the elderly Chinese people’s self-reported health. The putative processes are further examined in this part from the viewpoints of the “social network effect” and the “family care effect.” We estimate the subsequent model:


(4) $$M_{ic}=m_{0}+m_{1}\mathrm{UCHEC}S_{ic}+m_{2}X_{ic}+\vartheta_{ic}$$

where *M_ic_* represents the mechanism variables, namely, emotional support, social adaptation, social environment perception, self-efficacy, and caring from children reported by respondent i who lives in community c. Among them, the first four variables are designed to test social network effects; the last variable is designed to test family care effect. *ϑ_ic_* is an error term. Other variables are defined in the same way as in Equation 1. The coefficient *m*_1_, which captures the causal effects of *UCHECS_ic_* on *M_ic_*, is of central interest to our study.

Table 5 reports the mechanism analysis results. The estimated coefficients in Columns (1) – (5) are all positive significant at least at the 10% level. The results in Columns (1) – (3) show that if older adults use CHECS, they are more likely to feel accompanied, more likely to adapt to the current social changes, and more likely to believe that changes in the current social environment are not disadvantageous to older adults, indicating that using CHECS can significantly reduce social isolation in older people (37). Column (4) demonstrates that using CHECS can significantly increase the likelihood of self – perceived usefulness to society. Column (5) provides evidence supporting using CHECS makes it easier for older adults to feel that their children care about them. Furthermore, according to existing research, less social isolation, more self-efficacy, and more caring from children can improve older people’s health (38, 39). In a word, our results show that using CHECS may indirectly influence residents’ self-reported health through social network effects and family care effect.

**Table 5:** Mechanism analysis.

	Social network effect				Family care effect
	Emotional support	Social adaptation	Social environment perception	Self-efficacy	Family care
	(1)	(2)	(3)	(4)	(5)
UCHECS	0.175*** (0.066)	0.084* (0.049)	0.142* (0.074)	0.155** (0.072)	0.092* (0.051)
1st Kleibergen-Paap rk Wald F statistic	480.086	580.098	553.489	542.140	583.670
Hansen J-test *p*-value	0.188	0.688	0.357	0.629	0.921
Observations	6,382	6,389	6,416	6,531	6,637

***Significant at the 1% level; **Significant at the 5% level; *Significant at the 10% level. Standard errors clustered at the community level are reported in parentheses. We included all control variables in column 4 of Table 2.

## 4. Discussion

Community-based home-based eldercare is a significant strategy to boost the efficiency of social care services in China against the backdrop of the decline in family care. One of the first comprehensive research to investigate the effect and mechanism of utilizing CHECS on the self-reported health of older persons is this one. Utilizing instrumental variables to address the endogeneity issue and a nationally representative sample, we discovered that utilizing CHECS significantly improves the self-reported health of Chinese old people. To further support the fulfillment of Healthy Aging and Active Aging, we advise the government to boost the supply of CHECS and enhance their spatial accessibility.

Based on the results of the heterogeneity analysis, using CHECS did not have a significant impact on the self-reported health of older adults who are aged under 80, without functional disabilities, had chronic diseases, had received education, and live in households with higher income levels. However, there was a significant positive effect for older people who are aged under 80, with functional disabilities, without chronic diseases, had not received education, and live in households with lower income levels. These results mean that the current CHECS can only meet the needs of older adults who have lower requirements for their life quality and long-term care, reflecting that the quantity and quality of elderly care services provided by the community are a little lower (32), and lacking of qualified health care professionals. Considering the fact that Chinese older people have more diversified demands and higher quality requirements for elderly care services (40), as well as older people have the problem of living with illness for a long time and 75% of Chinese older people aged 60 or above suffer from at least one chronic disease (41, 42). We suggest the government should increase multi-level elderly care services supply and improve community service quality, especially health care services.

Additionally, we found that using CHECS had a substantial influence on older adults who reside in the center urban sections of city/county regions, but not on those who reside elsewhere in terms of self-reported health. We propose that more support be given towards developing home and community care services in rural areas, thereby enhancing their general well-being, taking into account that nearly half of China’s older adults live in rural areas (43) and older adults in rural areas have a stronger demand for these services (40).

Besides the above conclusions, heterogeneity analysis results identified that the use of CHECS had a significantly positive effect on self-reported health of older adults living alone and living with others, often interacting with their children and seldom interacting with their children, and a more pronounced positive effect on older adults who lived alone, and seldom interacting with their children. These results showed that using CHECS can effectively mitigate the negative effects of empty-nest on the health of older adults.

Based on our findings, we made the case that using CHECS can indirectly affect the self-reported health of Chinese elderly citizens through the “social network effect” and “family care effect.” To be more precise, the “social network effect” referring to the use of CHECS makes older people more likely to feel accompanied, more likely to adapt to the current social changes, less likely to feel that the changes to their social environment were detrimental to them, and more likely to feel useful to society. The “family care effect” referring to the use of CHECS greatly increases the likelihood that older people believe their offspring have always cared about them, which suggests that utilizing CHECS significantly boosts older people’s chances of interacting with younger people (44).

## 5. Conclusion

Based on 2018 CLASS data, we empirically estimated the causal impact of using CHECS on the self-reported health of Chinese older adults. We constructed a 2SLS model and exploited “the perceived rate of CHECS at the community level” and “except for individuals, the utilization rate of CHECS at the community level” as instrumental variables, which effectively addresses the endogeneity problem. The results show that older adults using CHECS can significantly improve their self-reported health, especially among people who under the age of 80, with functional disabilities, without chronic diseases, have not received education, live in households with lower income levels, live alone, seldom interact with their children, and live in the central urban regions of city/county regions. Moreover, this paper examined the possible mechanism. The results show that older adults using CHECS through “social network effect” and “family care effect” to improve their self-reported health.

However, there are certain limitations in our study. Firstly, due to the limitations of cross-sectional data, we could not discuss the long-term effect of using CHECS on the self-reported health of older adults. Secondly, although we have dealt with the endogeneity problems by using the 2SLS model, we have not distinguished the categories of CHECS. Different types of CHECS used by older adults may have different impacts on their health, this aspect can be further explored in the future.

## Data availability statement

The data analyzed in this study is subject to the following licenses/restrictions: data can be provided by the corresponding author on request. Requests to access these datasets should be directed to bjwei@sdfmu.edu.cn.

## Author contributions

YH: Conceptualization, Data curation, Investigation, Project administration, Supervision, Formal analysis, Methodology, Software, Writing – original draft, Writing – review & editing. BW: Funding acquisition, Resources, Validation, Visualization, Supervision, Writing – original draft, Writing – review & editing. YL: Project administration, Supervision, Writing – review & editing.

## Funding

The author(s) declare financial support was received for the research, authorship, and/or publication of this article. This work was supported by Research program of Medical and Health Science and Technology Development Plan Project of Shandong province (no. 202103070653).

## Conflict of interest

The authors declare that the research was conducted in the absence of any commercial or financial relationships that could be construed as a potential conflict of interest.

## Publisher’s note

All claims expressed in this article are solely those of the authors and do not necessarily represent those of their affiliated organizations, or those of the publisher, the editors and the reviewers. Any product that may be evaluated in this article, or claim that may be made by its manufacturer, is not guaranteed or endorsed by the publisher.
